# Pathological Aspects of COVID-19 as a Conformational Disease and the Use of Pharmacological Chaperones as a Potential Therapeutic Strategy

**DOI:** 10.3389/fphar.2020.01095

**Published:** 2020-07-10

**Authors:** Tomohiko Aoe

**Affiliations:** ^1^ Pain Center, Teikyo University Chiba Medical Center, Ichihara, Japan; ^2^ Department of Medicine, Teikyo University, Tokyo, Japan

**Keywords:** SARS-CoV-2, COVID-19, ER stress, unfolded protein response (UPR), apoptosis, proteostasis, pharmacological chaperone, BiP

## Abstract

Coronavirus disease 2019 (COVID-19), the seventh human coronavirus infectious disease, was first reported in Wuhan, China, in December 2019, followed by its rapid spread globally (251,059 deaths, on May 5, 2020, by Johns Hopkins University). An early clinical report showed that fever, cough, fatigue, sputum production, and myalgia were initial symptoms, with the development of pneumonia as the disease progressed. Increases in the level of serum liver enzymes, D-dimer, cardiac troponin I, and creatinine have been observed in severely ill patients, indicating that multiple organ failure had occurred in these cases. Lymphopenia and an increase in interleukin-6 (IL-6) were also observed. Although COVID-19 patients are administered glucocorticoid therapy to treat the excessive immune response to severe acute respiratory syndrome coronavirus-2 (SARS-CoV-2) infection, the efficacy of this form of therapy is unclear. Viremia is observed in severe cases, suggesting that in addition to type II alveolar epithelial cells, many cell types, such as vascular endothelial cells, cardiomyocytes, renal tubular cells, neuronal cells, and lymphocytes, may be damaged. The improvement of survival rates requires elucidation of the mechanism by which cellular damage occurs during viral infection. Cellular therapy, along with organ support systems such as oxygen therapy, artificial ventilation, extra corporeal membrane oxygenation and dialysis, as well as antiviral therapy, are required. Viral replication in infected host cells may perturb protein folding in the endoplasmic reticulum (ER), causing ER stress. Although an adaptive cellular response, i.e. the unfolded protein response, can compensate for the misfolded protein burden to some extent, continued viral proliferation may induce inflammation and cell death. Therefore, we propose that proteostasis dysfunction may cause conformational disorders in COVID-19. The application of pharmacological chaperone therapy to treat COVID-19 patients is additionally discussed.

## Introduction

The severe acute respiratory syndrome coronavirus-2 (SARS-CoV-2), which causes coronavirus disease 2019 (COVID-19), belongs to the genus *Betacoronavirus* of human coronaviruses (Wu et al., 2020; Zhou et al., 2020b); SARS-CoV-2 and the closely related SARS-CoV belong to lineage B, while the Middle East respiratory syndrome coronavirus (MERS-CoV) belongs to lineage C. SARS-CoV and MERS-CoV have caused severe respiratory disorders with higher mortality rates (Fung and Liu, 2019b).

SARS-CoV-2 is an enveloped virus whose genome comprises a single-stranded RNA molecule of 29,903 bases (Wu et al., 2020). The virus is composed of the RNA genome, a lipid membrane, and four constituent proteins, namely the spike glycoprotein, nucleocapsid protein, membrane protein, and envelope protein (Wu et al., 2020). As in SARS-CoV, the spike glycoprotein, membrane protein, and envelope protein are synthesized by the ribosome and inserted into the endoplasmic reticulum (ER) membrane during the replication of SARS-CoV-2. The newly synthesized polypeptides interact with ER molecular chaperones such as calnexin (Fukushi et al., 2012) to ensure correct protein folding. Mature viral proteins are transported from the ER to the ER-Golgi intermediate compartment (Masters, 2006), where viral particles are assembled from these proteins and the nucleocapsid protein, RNA, and lipid membrane (Fung and Liu, 2019b). Viral particles are transported along the secretory pathway to the plasma membrane, and secreted from the cell. Among the proteins that form SARS-CoV-2 viral particles, the spike glycoprotein, with a large molecular weight of 1,273 amino acids, exhibits N-linked glycosylation and plays an important role in mediating SARS-CoV-2 entry into host cells and the ensuing cellular damage (Walls et al., 2020; Zhou et al., 2020b).

SARS-CoV-2 enters epithelial cells in the mucosal membrane of the eyes, nose, and mouth, or the respiratory tract. As in SARS-CoV infection, during viral entry, the spike glycoprotein of SARS-CoV-2 attaches to the angiotensin-converting enzyme 2 (ACE2) protein, which is expressed on the cell surface of host cells, and uses it as a receptor (Walls et al., 2020; Zhou et al., 2020b). The SARS-CoV-2 spike glycoprotein is primed by the host serine protease, TMPRSS2, which confers the ability to bind to ACE2 (Hoffmann et al., 2020). ACE2 is expressed in various organs, such as the oral and nasal mucosa, nasopharynx, lung, stomach, small intestine, colon, skin, lymph nodes, thymus, bone marrow, spleen, liver, kidney, and brain (Hamming et al., 2004). It has been suggested that the spike glycoprotein recognizes molecules other than ACE2 expressed on the cell surface. A structural analysis of the spike glycoprotein of SARS-CoV-2 suggests that this protein can additionally bind to glucose-regulated protein 78 (GRP78) on the cell surface (Ibrahim et al., 2020). GRP78 is also known as binding immunoglobulin protein (BiP), an ER chaperone.

## ER Stress and the UPR

**Figure 1:**
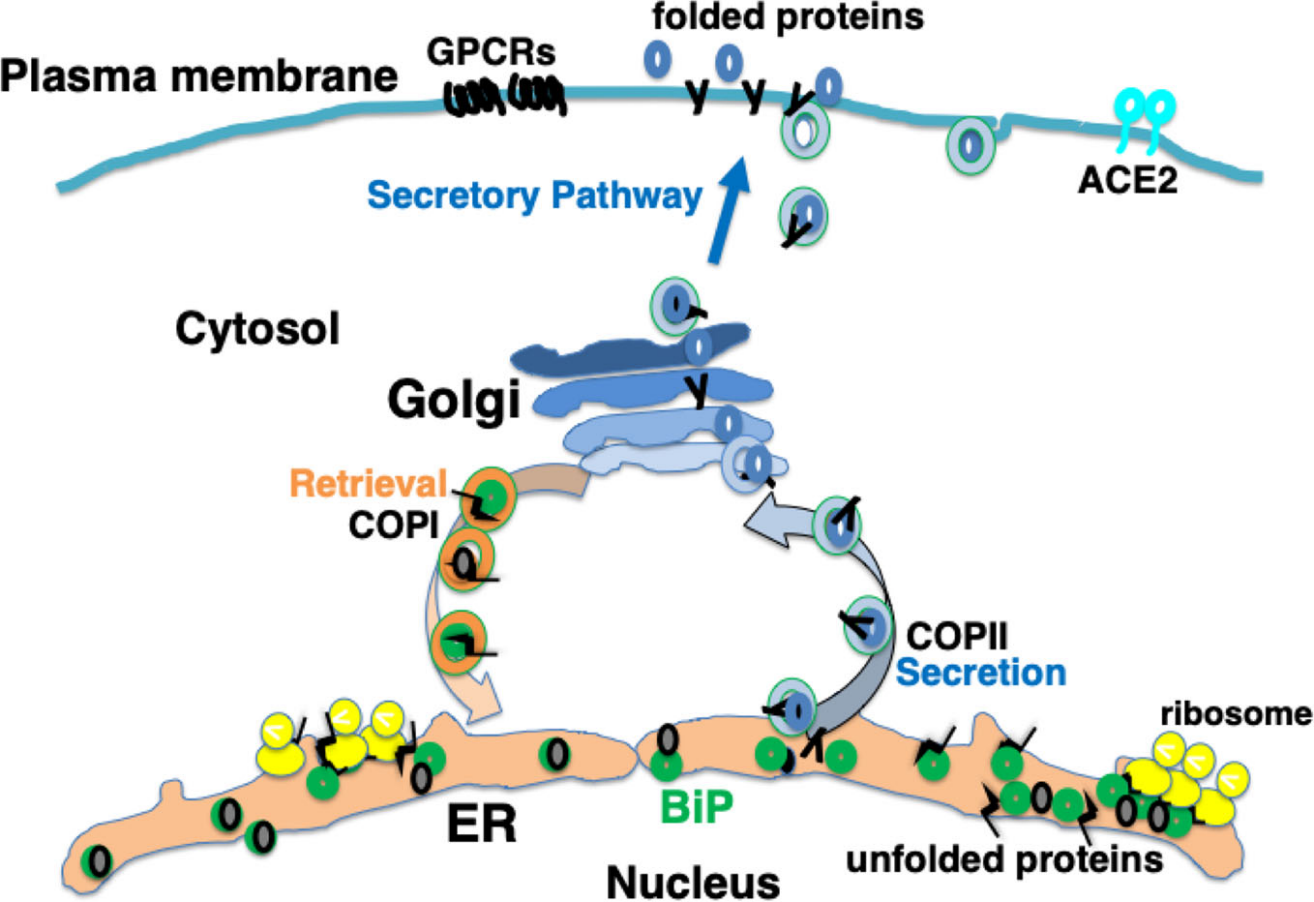
Newly synthesized polypeptides are inserted into the endoplasmic reticulum (ER). Properly folded proteins are transported to the secretory pathway. Interaction with ER chaperones such as BiP facilitates proper folding of nascent proteins. Mature proteins are released into the secretory pathway *via* coat protein II (COPII) vesicular transport. A certain fraction of KDEL proteins, such as BiP, associating with unfolded proteins are secreted; these are retrieved *via* KDEL receptors from the cis-Golgi to the ER through COPI vesicular transport.

Secretory proteins and membrane proteins are synthesized by ribosomes on the ER membrane and inserted into the ER. These nascent polypeptides interact with ER molecular chaperones such as BiP, protein disulfide isomerase (PDI), calreticulin, and calnexin to undergo correct folding, sugar chain modification, and complex formation (Wang and Kaufman, 2016; Rapoport et al., 2017). Properly folded proteins are secreted from the ER by coat protein complex II (COPII) vesicles to the Golgi apparatus, and then transported to the plasma membrane. Proteins such as hormones are secreted from the cell, while others, such as G protein coupled receptors (GPCRs), are expressed on the plasma membrane as cell-surface receptors (Peotter et al., 2019) (**Figure 1**).

**Figure 2:**
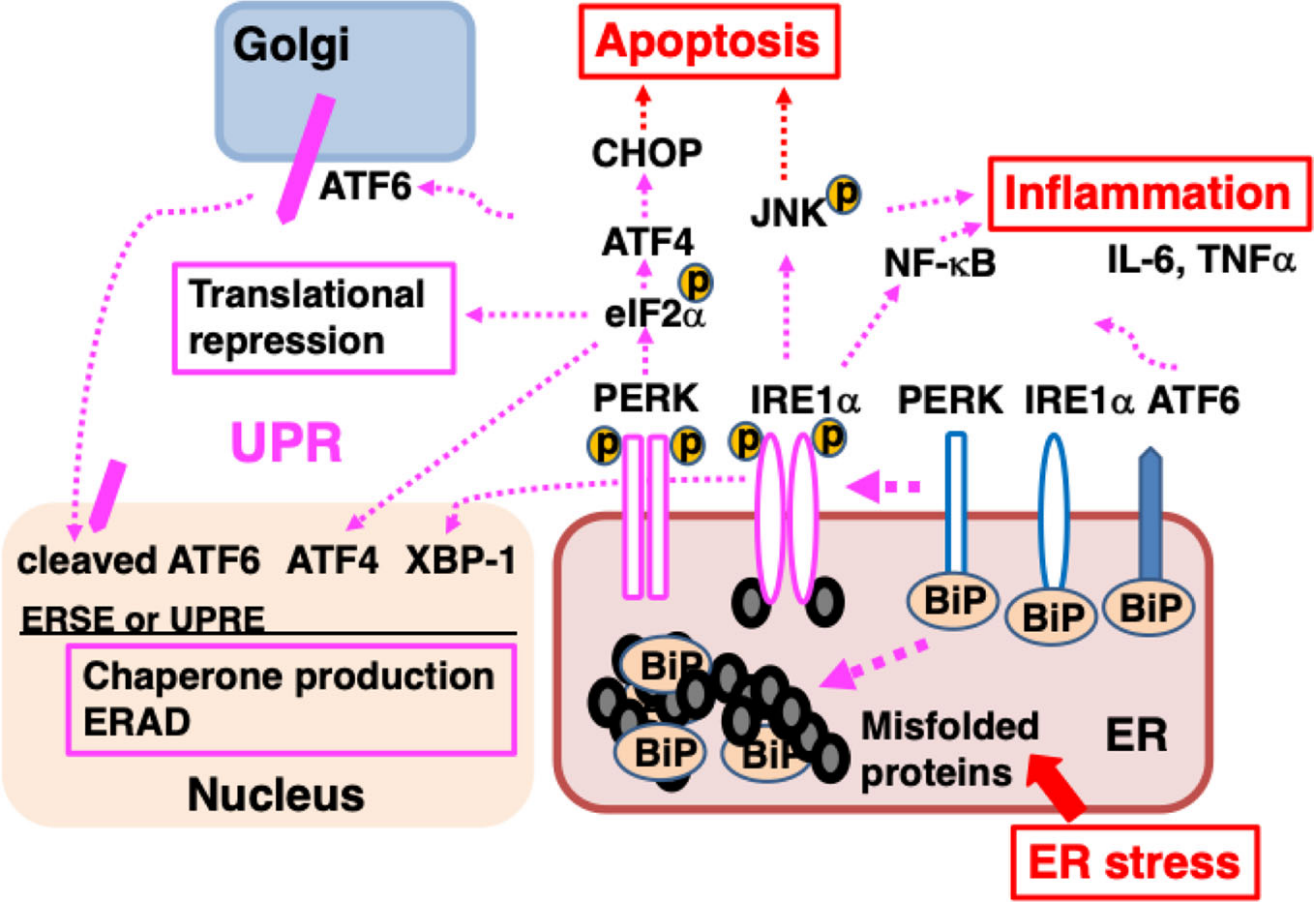
ER stress causes the accumulation of unfolded proteins in the ER, inducing the unfolded protein response (UPR). Misfolded proteins accumulate in the ER when protein folding is disturbed due to ER stress such as intrinsic defects (e.g., mutated sequences, missing subunits) or extrinsic injuries (e.g., ischemia, malnutrition, hypoxia, and toxicity). BiP dissociates from ATF6, IRE1α, and PERK, and associates with misfolded proteins; dissociation from BiP initiates the UPR. ATF6 is transported to the Golgi, where it is cleaved and the cytoplasmic portion of ATF6 is transported to the nucleus; there, it functions as a transcription factor, binding to ER stress response element (ERSE) and promoting the transcription of genes important for the UPR. After dissociating from BiP, IRE1α, and PERK multimerize and are activated by autophosphorylation. IRE1α is a type I membrane protein containing a serine/threonine kinase domain and an endoribonuclease domain at its cytoplasmic carboxyl terminus. Activated IRE1α splices XBP1 mRNA. The XBP1 protein binds to UPR elements (UPREs) in the transcriptional region of various genes required for the UPR and promotes their transcription. PERK is a serine/threonine kinase that phosphorylates and inactivates eIF2α, thereby halting the translation of most proteins. However, the translation of ATF4 is induced. ATF4 acts as a transcription factor. The UPR enhances the ability of cells to deal with increased levels of misfolded proteins through chaperone production, translational repression, and ER-associated protein degradation (ERAD). Activation of IRE1α leads to inflammation through the activation of MAP kinases (JNK) and NF-κB, inducing elevated expression of pro-inflammatory cytokines, such as TNFα and IL-6. Persistent ER stress induces the expression of CHOP, leading to apoptosis.

BiP is a major ER molecular chaperone; most of them are distributed to the ER lumen by binding to ER membrane proteins such as activated transcription factor 6 (ATF6), inositol requiring kinase-1 (IRE1), and PKR-like ER related kinase (PERK). BiP additionally binds unfolded nascent polypeptides to ensure proper folding without the formation of aggregates (Hendershot, 2004). External factors such as ischemia, oxidative stress, malnutrition, and exposure to toxic substances, as well as internal factors such as genetic abnormalities resulting in the production of mutant proteins, disrupt protein folding in the ER. The accumulation of misfolded proteins that are unable to form proper three-dimensional structures or complexes in the ER causes ER stress, leading to an adaptive cellular response known as the unfolded protein response (UPR) (Hetz and Papa, 2018). BiP binds to misfolded proteins and dissociates from ATF6, IRE1, and PERK, resulting in the activation of these membrane proteins. ATF6 is a type 2 transmembrane protein that is transported from the ER to the Golgi complex, where it undergoes cleavage of its cytoplasmic amino-terminal side to become a transcription factor. ATF6 translocates to the nucleus and induces the transcription of genes involved in the UPR such as those that encode ER molecular chaperones (Shen et al., 2002). IRE1 and PERK form a homo-multimer and are activated by autophosphorylation. IRE1, which exhibits RNase activity, splices X-box binding protein-1 (XBP-1) mRNA. The spliced form is translated into XBP-1 protein, which acts as a transcription factor and mediates progression of the UPR (Lee et al., 2002). IRE1 also activates mitogen-activated protein (MAP) kinases such as c-jun N-terminal kinase (JNK) (Urano et al., 2000), triggering inflammation (Hasnain et al., 2012), promoting autophagy (Fung and Liu, 2019a; Sozen et al., 2020). PERK phosphorylates eukaryotic initiation factor 2α (eIF2α), leading to the suppression of general protein translation, which reduces the load of newly synthesized unfolded polypeptides on the ER (Hughes and Mallucci, 2019). The UPR promotes increased production of cytoprotective molecules such as ER molecular chaperones, suppression of the protein translation of most proteins, accelerated degradation of misfolded proteins (ER-associated degradation; ERAD) (**Figure 2**).

Thus, the capacity of cells to handle misfolded proteins in the ER is expanded, and cells can tolerate ER stress to a certain degree (Hetz and Papa, 2018). However, in response to excessive load of misfolded proteins due to a greater invasion like a higher viral load, the UPR is prolonged. The activation of PERK induces the expression of ATF4, leading to the production of the transcription factor C/EBP homologous protein (CHOP), which induces the downregulation of anti-apoptotic Bcl-2 and results in apoptotic cell death (Oyadomari and Mori, 2004; Rozpedek et al., 2016). Persistent ER stress alleviates Ca^2+^ homeostasis. The ER comprises a major pool of Ca^2+^, and ER molecular chaperones such as BiP are capable of binding Ca^2+^. ER stress induces the outflow of Ca^2+^ from the ER and the influx of Ca^2+^ into mitochondria, leading to the release of cytochrome C and the activation of caspases 9 and 3, resulting in apoptosis, cell death (Ye et al., 2008; Bahar et al., 2016).

## Proteostasis and Conformational Diseases

The proteostasis network, which is mediated by the interaction with ER molecular chaperones and cytosolic molecular chaperones such as heat shock protein 70 (HSP70), serves as a quality control process for ensuring proper protein folding and complex assembly. Failure of proteostasis leads to the accumulation of misfolded proteins, which triggers a persistent integrated stress response (ISR), including the UPR in the ER and heat shock response in the cytosol (Labbadia and Morimoto, 2015).

The accumulation of misfolded proteins and ER stress are associated with neurodegenerative diseases such as Alzheimer's disease (Ferreira and Klein, 2011), Parkinson's disease (Gibb and Lees, 1988), amyotrophic lateral sclerosis (ALS) (Rosen et al., 1993), and prion disease (Park et al., 2017), as well as other diseases such as cardiomyopathy (Hamada et al., 2004), pulmonary fibrosis (Peca et al., 2015), chronic renal tubular disease (Kimura et al., 2008), diabetes (Ozcan et al., 2006), obesity (Shan et al., 2017), and aging in human and animal models (Labbadia and Morimoto, 2015). Acute diseases such as respiratory failure and ischemia-reperfusion injury additionally involve the induction of ER stress and the UPR (Tajiri et al., 2004; Mimura et al., 2007; Klymenko et al., 2019).

Owing to genetic mutations, some proteins may evade the proteostasis network, causing protein aggregation both within and outside of cells. Alpha 1-antitrypsin deficiency (AATD) is a genetic disease caused by a mutation in the *SERPINA1* gene (serine proteinase inhibitor, group A, member 1), which encodes a secretory protein, alpha 1-antitrypsin (AAT). The Z mutant of AAT, which is the most common variant of this protein in AATD, accumulates in the ER of hepatocytes without undergoing proper folding; this leads to ER stress and cellular dysfunction. In addition, AAT inhibits the activity of neutrophil serine proteases in blood. Thus, the deficiency of properly folded AAT causes the activation of neutrophil serine proteases, resulting in lung injury (Karatas and Bouchecareilh, 2020). Cystic fibrosis, another example of a genetic disorder, is caused by mutations in the cystic fibrosis transmembrane conductance regulator (CFTR) protein, which is a chloride ion channel expressed on the epithelial cells of the lung, pancreas, and other organs. The most common mutation of the *CFTR* gene, F508del results in the translation of a mutant CFTR, which exhibits impaired trafficking (Cheng et al., 1990). The loss of cell-surface expression of CFTR results in the production of thick mucus and clog formation. Accumulation of the F508del mutant CFTR in the ER induces ER stress, causing cellular dysfunction and inflammation in airway epithelia (Ribeiro and Boucher, 2010) and production of interleukin-6 (IL-6) and tumor necrosis factor alpha (TNFα) by innate immune cells (Lara-Reyna et al., 2019).

Pulmonary surfactant, which is composed of phospholipids and the pulmonary surfactant proteins (SP)-A, -B, -C, and -D, reduces alveolar surface tension to facilitate spontaneous physiological respiration. The SP-C precursor contains the BRICHOS domain, which prevents amyloid formation (Dolfe et al., 2016). Several mutations in the BRICHOS domain of the *SP-C* gene have been reported to induce protein aggregation and ER stress, resulting in cell death and chronic interstitial lung diseases (Beers and Mulugeta, 2005; Peca et al., 2015). Owing to their native structure, some proteins, such as olfactory GPCR, are difficult to fold properly. GPCR is a membrane protein with a highly complex structure that penetrates the membrane seven times; because of this complex structure, nascent polypeptides, despite having normal gene sequences, may not all undergo proper folding. Many nascent polypeptides may be misfolded, retained in the ER, and degraded by ERAD and autophagy (Lu et al., 2003; Lu et al., 2004).

Diseases related to the dysfunction of proteostasis, which are known as conformational diseases (Carrell and Lomas, 1997; Kopito and Ron, 2000), occur in various cell types and are characterized by organ-specific symptoms corresponding to them. Although such conformational diseases are sometimes caused by a mutation in the gene encoding a specific protein, resulting in aggregation of the misfolded mutant protein, they can also be caused by dysfunction of the proteostasis system itself. BiP is a luminal chaperone present in the ER; a fraction of BiP proteins associating with unfolded proteins, are secreted from the ER by COPII vesicles (Raykhel et al., 2007). At the Golgi apparatus, the Lys-Asp-Glu-Leu (KDEL) carboxyl terminal of BiP is recognized by the KDEL receptor, which facilitates the return of BiP to the ER *via* COPI vesicles. The KDEL-retrieval system is part of the proteostasis network (Yamamoto et al., 2001). Homozygous knock-in mice expressing mutant BiP with the KDEL amino acid sequence deleted, instead of normal BiP, are born according to Mendelian law. As a result, all homozygous mutant BiP mice reportedly died from neonatal acute respiratory distress syndrome (ARDS) on the first day after birth (Mimura et al., 2007). In the type II alveolar epithelial cells of these mice, folding of surfactant proteins was impaired, leading to the loss of the secretion of pulmonary surfactant. In addition, the accumulation of misfolded surfactant proteins in the ER causes ER stress, inducing the expression of CHOP and triggering apoptosis. The proper assembly of correctly folded individual surfactant proteins is required for the production and secretion of pulmonary surfactant, which enables breathing by keeping the alveolar space open. Proteostasis therefore plays an important role in the production and secretion of pulmonary surfactant.

## Chronic Comorbidities and Proteostasis

Sporadic neurodegenerative diseases often accompany aging (Labbadia and Morimoto, 2015). Heterozygous mutant BiP mice are capable of survival, and their lifespan is not significantly different from that of wild-type mice. However, in addition to wild-type BiP, these mice also express mutant BiP lacking the KDEL sequence. Misfolded proteins bound to the mutant BiP may escape from the proteostasis network without being recognized by the KDEL receptor at the Golgi complex, even if secreted from the ER (Jin et al., 2017). In aged mutated BiP mice, ubiquitinated protein aggregation was observed in the spinal cord and brain (Jin et al., 2014). Motor and cognitive dysfunction were additionally observed (Jin et al., 2018). With aging, organ symptoms such as renal tubular injury were also found (Kimura et al., 2008). Attenuation of proteostasis can lead to acute cellular dysfunction and cell death, as well as chronic cellular dysfunction and organ failure. The function of ER chaperones that play an important role in the proteostasis network declines with aging (Brown and Naidoo, 2012).

Preclinical studies suggest that vascular endothelial cells, cardiomyocytes, alveolar epithelial cells, neuronal cells, and lymphocytes may already be subjected to ER stress in individuals with comorbidities such as hypertension (Hasty and Harrison, 2012; Young, 2017; Ochoa et al., 2018), diabetes (Back and Kaufman, 2012), obesity (Chen et al., 2016), chronic obstructive pulmonary diseases (Liu et al., 2018), and neurodegenerative diseases (Labbadia and Morimoto, 2015). Obesity causes chronic inflammation in adipocytes as well as macrophages that induces elevated expression of pro-inflammatory cytokines, TNFα, and IL-6 (Solinas et al., 2007). Alleviation of ER stress by pharmacological chaperones suppresses NF-κB activity and inflammation in diet-induced obese mice (Chen et al., 2016).

**Figure 3:**
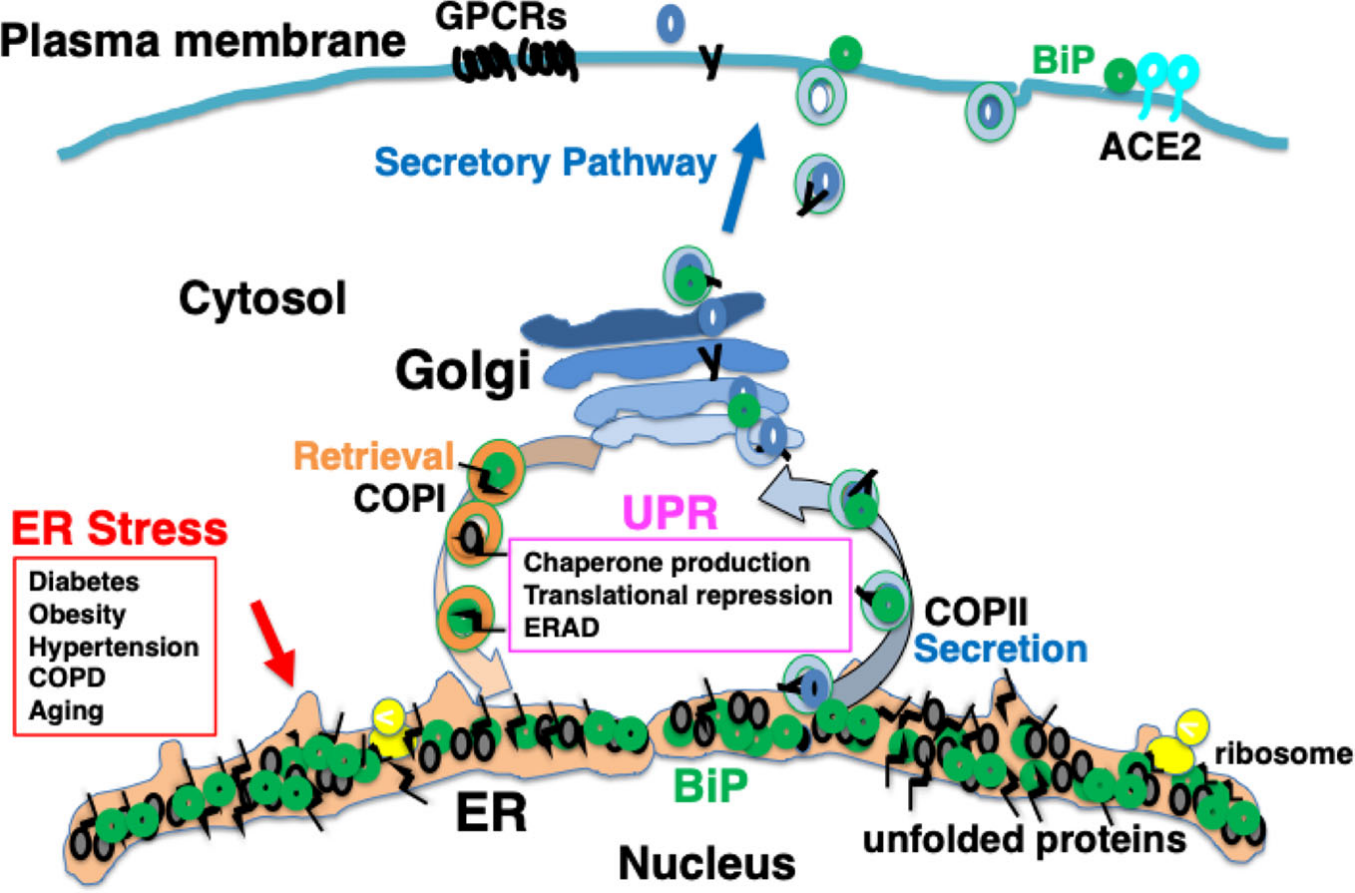
Chronic comorbidity is associated with ER stress. Among patients with complications and elderly people, protein folding in the ER is impaired to a certain extent; therefore, the ability of the UPR to accommodate further ER stress may be limited.

It is considered that among patients with complications and elderly people, protein folding in the ER is impaired to a certain extent; therefore, the ability of the UPR to accommodate further ER stress may be limited. As a consequence, proteostasis in such individuals may be sensitive to further stress in the ER, for example, in conditions such as viral protein overload (Hrincius et al., 2015; Guan et al., 2020; Zhou et al., 2020a) (**Figure 3**).

## SARS-COV, SARS-CoV-2, and ER Stress

The organization of the genome of SARS-CoV-2 is closely related to that of SARS-CoV, and the structures of the proteins of both viruses are similar (Wu et al., 2020). Furthermore, both SARS-CoV and SARS-CoV-2 recognize the same ACE2 receptor expressed on host cells, and viral entry is mediated *via* their spike glycoproteins (Walls et al., 2020; Zhou et al., 2020b). Both viruses utilize the protein translation machinery of host cells for their replication. The spike glycoprotein, membrane protein, and envelope protein of SARS-CoV and SARS-CoV-2 are inserted into the ER after translation by ribosomes, where they undergo protein folding and glycosylation. Preclinical studies on SARS-CoV and other viruses have revealed that a result of rapid viral replication, nascent unfolded viral polypeptides accumulate in the ER, possibly causing ER stress (Huang et al., 2016).

In SARS-CoV infected patients, apoptosis in pneumocytes, T and B lymphocytes, and monocytes has been found to occur in the lung, spleen, and lymph nodes (Zhang et al., 2003). Lymphopenia and reduced counts of CD4-positive and CD8-positive T cells have been observed early in the disease course in SARS patients (Wong et al., 2003). When SARS-CoV was expressed in cultured cells, the spike glycoprotein accumulated in the ER and induced the UPR (Chan et al., 2006). In addition to the transcription of molecular chaperones such as BiP and GRP94, the expression of CHOP was also induced. This study suggested that when SARS-CoV induced the UPR, it did not activate ATF6, instead preferentially triggering the activation of PERK, leading to CHOP induction through the phosphorylation of eIF2α and the activation of transcription factor ATF4. Accumulation of the accessory 3α protein in the ER was found in SARS-CoV-infected cultured cells, where the activation of PERK, leading to the induction of ATF4 and CHOP were additionally observed (Minakshi et al., 2009). The SARS-CoV open reading frame (ORF) 8b protein has also been found to undergo aggregation in infected cells, and induce ER stress (Shi et al., 2019). These clinical observations in SARS patients and preclinical studies of SARS-CoV suggests that SARS-CoV infection induces ER stress and subsequent apoptosis in infected cells.

Several studies suggest that ACE2-mediated signaling reduces the effects of ER stress (Zhang et al., 2018). Stimulation of ACE2 by the ligands angiotensin 1–7 suppresses apoptosis mediated by ER stress in alveolar epithelial cells with accumulation of mutant surfactant protein C (Uhal et al., 2013). Shedding of ACE2 by spike glycoproteins may exacerbate ER stress. Individuals with diseases such as asthma and chronic obstructive pulmonary disease have similar levels of ACE2 expression to healthy people, but cigarette smokers have the elevated expression (Li et al., 2020a). It is unclear whether people with these comorbidities are at increased risk of SARS-CoV-2 infection based on their ACE2 expression levels.

**Figure 4:**
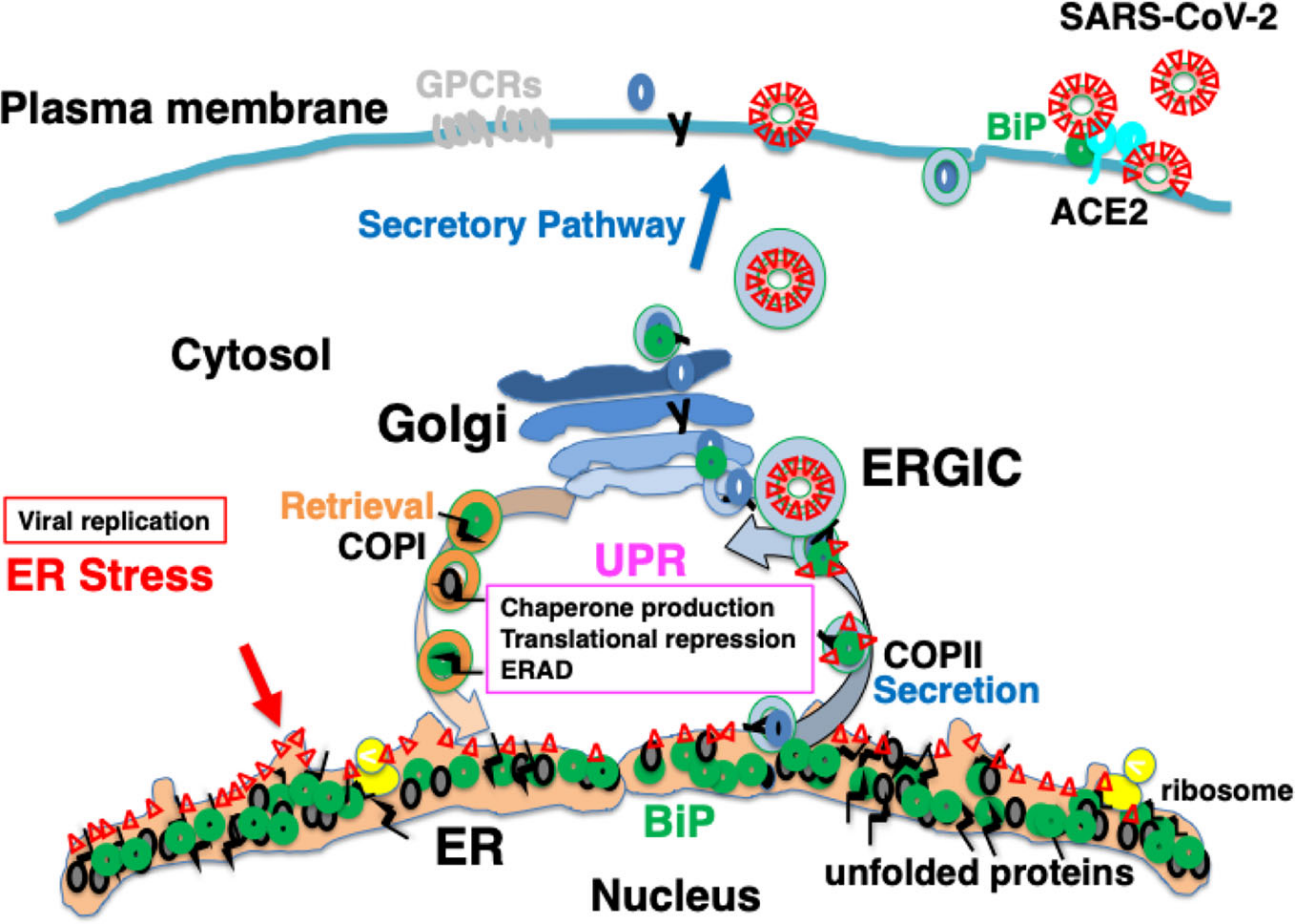
Infection with severe acute respiratory syndrome coronavirus-2 (SARS-CoV-2) may cause ER stress, inducing an adaptive response known as the UPR. SARS-CoV-2 infects ACE2-expressing cells. BiP on the cell surface may increase infectability as observed in Middle East respiratory syndrome coronavirus (MERS-CoV) infection. Mature viral proteins are transported from the ER to the ER-Golgi intermediate compartment (ERGIC), where viral particles are assembled from these proteins as well as nucleocapsid proteins, RNA, and lipid membranes. The replication of viral proteins causes ER stress, which is tolerated by the cell because of the UPR. Unstable proteins such as GPCRs, like olfactory receptors and taste receptors, may be susceptible to ERAD.

The spike glycoprotein of MERS-CoV recognizes dipeptidyl peptidase 4 (DPP4) as a receptor; DPP4 bound to BiP is recognized more strongly than DPP4 alone, resulting in enhanced infectivity (Chu et al., 2018). Under ER stress, the UPR induces the production of BiP, which saturates reverse transport by the KDEL receptor. This is considered to result in missorting of BiP from the Golgi to the cell surface (Kokubun et al., 2019). Some BiP molecules are secreted extracellularly, whereas others are attached to the cell membrane *via* the proline-rich region near the carboxyl terminus (Tseng et al., 2019). BiP expressed on the plasma membrane may be involved in signal transduction as a cell-surface receptor (Tsai et al., 2018). The spike glycoprotein of SARS-CoV-2 may recognize BiP on the cell surface (Ibrahim et al., 2020); therefore, ER stress may increase infectivity in patients with various comorbidities and elderly individuals (**Figure 4**).

## COVID-19 as a Conformational Disease

SARS-CoV-2 entry occurs through the mucous membranes of the eyes, nose, and mouth. This is followed by the appearance of symptoms such as fever, cough, fatigue, sputum production, shortness of breath, and myalgia (Guan et al., 2020; Zhou et al., 2020a), although many infected people do not show clear symptoms (Arons et al., 2020). Some people complain of abnormal taste and smell as initial symptoms (Xydakis et al., 2020). Smell is sensed by various olfactory receptors expressed on the surface of olfactory cells. Olfactory receptors are GPCRs, which have a complex structure that penetrates the membrane seven times. As a result, nascent peptides of olfactory receptors show high rates of misfolding, and misfolded peptides are retained in the ER and degraded by ERAD and autophagy (Lu et al., 2003; Lu et al., 2004). Taste is sensed by taste receptor type 1 (T1R) and taste receptor type 2 (T2R) receptors, which are also seven-transmembrane GPCR proteins expressed in taste cells distributed in the taste buds of the tongue, soft palate, and epiglottis (Nuemket et al., 2017). The folding of these receptor proteins is considered susceptible to impaired proteostasis by SARS-CoV-2 infection.

The most common fatal complication in COVID-19 patients is pneumonia, followed by ARDS (Guan et al., 2020; Huang et al., 2020; Yang et al., 2020; Zhou et al., 2020a). Some patients are not aware of hypoxemia (Ottestad et al., 2020). Among COVID-19 patients with CT findings of pneumonia, those without other symptoms such as fever and cough have been reported (Zhang et al., 2020). This asymptomatic silent pneumonia may be caused by impaired synthesis of pulmonary surfactant in type II alveolar epithelial cells infected with SARS-CoV-2 rather than inflammation.

In critically ill patients, serum levels of cytokines such as IL-6 (Zhou et al., 2020a), IL-2, IL-7, IL-10, GCSF, IP10, MCP1, MIP1A, and TNFα (Huang et al., 2020) are elevated. Pneumonia and ARDS in COVID-19 are considered to result from excessive systemic inflammation, a severe cytokine storm; therefore, corticosteroids are administered to these patients (Guan et al., 2020; Huang et al., 2020; Zhou et al., 2020a). However, the clinical data for SARS, MERS, and COVID-19 do not support the clinical efficacy of corticosteroids (Stockman et al., 2006; Russell et al., 2020). Complications such as avascular necrosis, delayed viral clearance, psychosis, and diabetes have been observed in SARS patients (Stockman et al., 2006; Russell et al., 2020). The World Health Organization does not recommend the use of corticosteroids for COVID-19 (World Health Organization, 2020). In COVID-19 patients with severe disease, low lymphocyte counts have been observed (Guan et al., 2020; Huang et al., 2020; Li et al., 2020b; Zhou et al., 2020a). Further, it remains unclear to what extent excessive systemic inflammation contributes to the cellular damage observed in patients with COVID-19.

**Figure 5:**
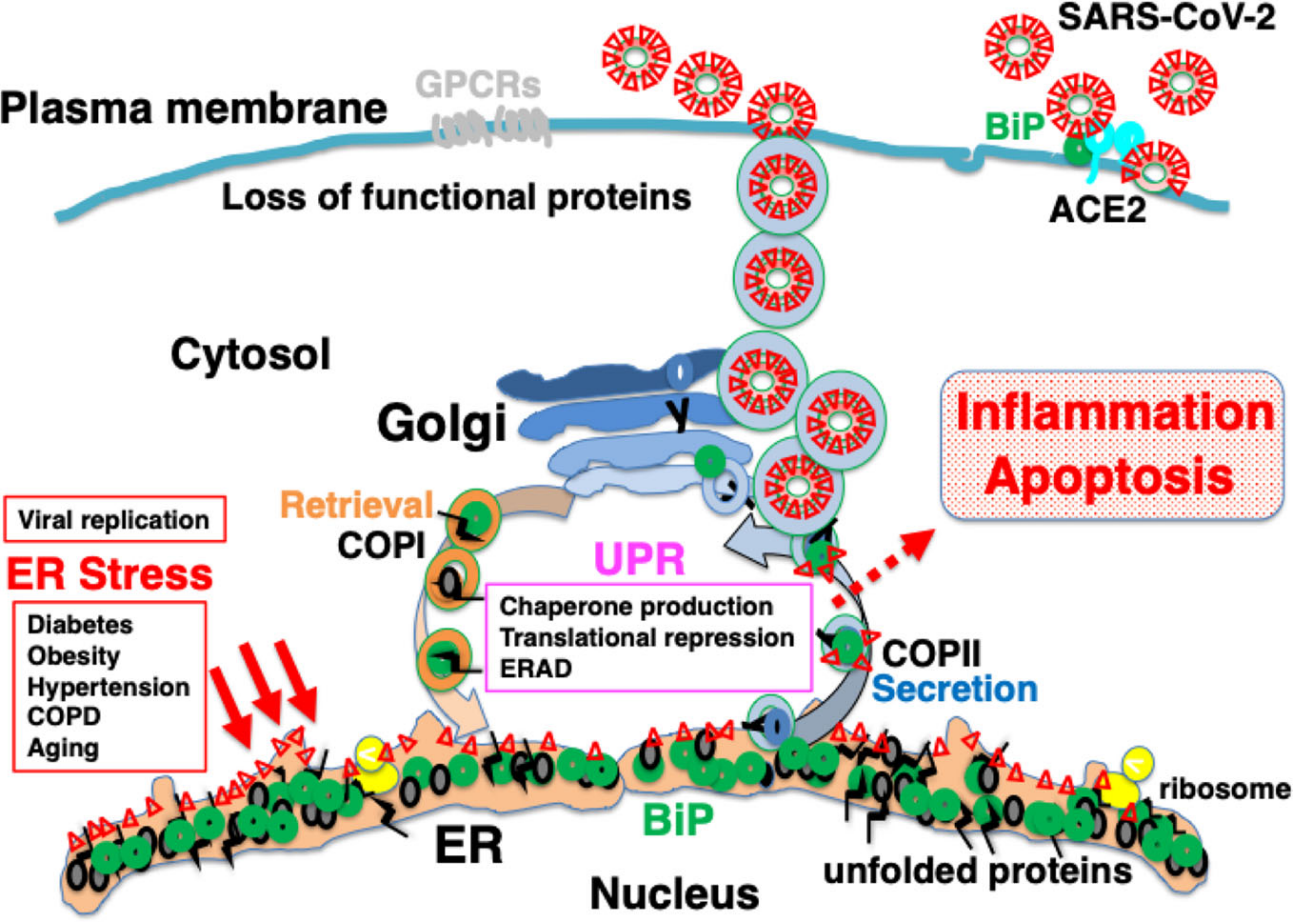
Explosive SARS-CoV-2 replication may induce persistent ER stress, leading to inflammation and apoptosis. The extremely rapid rate of replication of SARS-CoV-2 increases ER stress to levels that cannot be accommodated by the UPR, leading to inflammation and apoptosis. Patients with comorbidities may have limited capacity for proteostasis, and be sensitive to ER stress caused by viral protein overload. Pharmacological chaperones may reduce ER stress.

An additional explanation for the etiology of COVID-19 is that the intracellular replication of SARS-CoV-2 itself is cytotoxic to host cells. SARS-CoV-2 infects ACE2-expressing cells such as type II alveolar epithelial cells, and proliferates by hijacking the protein translational machinery of host cells. The spike glycoprotein, membrane protein, and envelope protein, which are the main constituent proteins of the novel coronavirus, are co-translationally inserted into the ER. The explosive production of nascent viral polypeptides may cause ER stress and induce the UPR. In most individuals, cells have the ability to tolerate ER stress caused by viral infection because of the UPR, which expands the capacity of proteostasis, enabling infected cells to survive (**Figure 4**). However, when the burden of viral protein replication cannot be accommodated by the UPR, the production of functional proteins in the host cells is suppressed and cellular function is impaired, eventually leading to apoptosis (**Figure 5**). The assembly and secretion of pulmonary surfactant in type II alveolar epithelial cells is sensitive to impaired proteostasis, and the loss of pulmonary surfactant secretion in addition to apoptosis of type II alveolar epithelial cells may cause ARDS. Ultrastructural study of lung tissues of COVID-19 patients revealed viral particles in cytoplasm of type II cells with markedly swollen mitochondria and dilated ER (Li et al., 2020b). Pyroptosis and apoptosis were observed in the lung cells. Massive infiltration of CD68+ macrophages and neutrophils in lung tissues accompanied with elevated serum cytokines including IL6, IL8, TNFα, IP10, MCP1, and RANTES were also observed (Li et al., 2020b). In SARS patients, the expression of pro-inflammatory cytokines such as MCP-1, TGF-β, TNF-α, IL-1β, and IL-6 was upregulated in SARS-CoV infected, ACE2-expressing cells such as alveolar epithelial cells and macrophages in the lung (He et al., 2006). Innate immunity activated by viral ER stress may contribute to the disease (Hrincius et al., 2015).

SARS-CoV-2 initially invades epithelial cells in the upper and lower airway, and viremia has been observed in patients with severe COVID-19 (Huang et al., 2020). ACE2 is expressed in multiple organs such as the kidney, testis, intestinal tract, and heart, as well as in vascular endothelial cells (Hamming et al., 2004). The explosive production of viral proteins in infected cells and the resulting elevated ER stress may impair proteostasis in host cells, inducing suppression of the translation of functional proteins, and triggering apoptosis. Increases in the levels of serum liver enzymes, D-dimer, cardiac troponin I, creatinine, and lymphopenia have been observed in severely ill patients (Zhou et al., 2020a). The Sequential Organ Failure Assessment (SOFA) score of patients who died from COVID-19 is reported to be high, indicating that these individuals had suffered multiple organ failure, including renal dysfunction, liver dysfunction, cardiovascular dysfunction, gastrointestinal disorders, neuronal disorders, and immune suppression (Guan et al., 2020; Mao et al., 2020; Zhou et al., 2020a). Pathological analysis of patients with COVID-19 indicated the presence of viral particles in vascular endothelial cells. Direct viral infection caused diffuse vascular endothelial inflammation in multiple organs including lung, heart, kidney, liver, and small intestine (Varga et al., 2020). The induction of caspase 3 and apoptotic bodies in endothelial cells and mononuclear cells in small bowel and lung specimens has been observed (Varga et al., 2020).

Although the removal of SARS-CoV-2 or infected cells would represent a “cure” for COVID-19, SARS-CoV-2 may continue to proliferate in infected cells in a state where cellular dysfunction is tolerable by the host's UPR. The duration of the resulting subclinical infection is unclear. It remains unknown whether chronic infection with SARS-CoV-2 may cause chronic conformational diseases in infected cells and organs such as interstitial pneumonia, tubulointerstitial renal fibrosis, and neurodegenerative disease.

## The Use of Pharmacological Chaperones as a Potential Therapeutic Strategy for COVID-19

Several drugs like Remdesivir, a nucleotide analogue prodrug that inhibits viral RNA polymerases seem to be promising for anti-SARS-CoV-2 therapy (Grein et al., 2020; Lythgoe and Middleton, 2020). If antiviral therapy is administered at the later stages of the infection, i.e. when viral propagation occurs and cell death is initiated, the effect may be limited, and cell death and mortality may be inevitable. Antiviral therapy should be administered prior to the onset of rapid viral replication. Surfactant replacement therapy may be effective in improving respiratory function (Hentschel et al., 2020). In addition, the therapeutic effect of pharmacological chaperones that promote protein folding in the ER, is considered to involve alleviation of ER stress, and suppression of cellular dysfunction, inflammation and apoptosis, and therefore represents a promising treatment strategy in COVID-19. Pharmacological chaperone therapy may additionally “buy more time” to effectively treat patients through antiviral therapy with infected cells alive.

Several candidate pharmacological chaperones, such as tauroursodeoxycholic acid (TUDCA) and 4-phenyl butyric acid (PBA), may offer promising therapeutic avenues in this context (Chen et al., 2016). TUDCA has been most well studied, preclinically and clinically, to date (Kusaczuk, 2019). TUDCA is a taurine conjugate of ursodeoxycholic acid (UDCA), which promotes bile acid secretion and exerts a hepatocyte-protective effect. Clinical use of UDCA for the treatment of primary biliary cholangitis has been approved by the U.S. Food and Drug Administration. Human bile acids contain UDCA; TUDCA/UDCA is also expected to be clinically applicable in the treatment of various diseases as a pharmacological chaperone that promotes protein folding in the ER (Vang et al., 2014). TUDCA is water-soluble, and can be administered orally or intravenously. It suppresses the dissociation of BiP from PERK, and inhibits the phosphorylation of eIF2α and induction of ATF4-CHOP. As a result, the expression of anti-apoptotic BCL-2 expression is increased, while that of pro-apoptotic Bax is decreased, and caspase-3 activation is suppressed (Rodrigues et al., 1999). The activity of pharmacological chaperones and the anti-apoptotic effect of TUDCA against acute conditions such as ischemia, hypoxia, and pressure overload on the cardiovascular system have been demonstrated in various preclinical studies using cultured cells and mouse models (Kusaczuk, 2019). Chronic ER stress due to obesity induces adipose inflammation and suppresses signaling from the insulin receptor to insulin receptor substrate-1 (IRS-1), leading to insulin resistance. TUDCA ameliorates these signaling abnormalities (Ozcan et al., 2006), and reduces adipose tissue inflammation (Chen et al., 2016). In neurodegenerative disease models such as prion disease and ALS, ER stress due to aggregated proteins in neuronal cells is relieved and neuronal cell death is reduced (Kusaczuk, 2019). TUDCA and PBA relieve influenza A virus-induced inflammation, and suppress the production of IL-6 in lung epithelial cells (Hrincius et al., 2015).

Several clinical trials of TUDCA for conformational diseases induced by ER stress have been reported (**Table 1**). ALS is a neurodegenerative disease in which unstable proteins, such as mutant Cu/Zn superoxide dismutase 1 (SOD1), aggregate in motor neurons, inducing cellular dysfunction and cell death, and resulting in motor disability. In one trial, TUDCA was orally administered to 14 patients at 2,000 mg daily for 54 weeks; TUDCA-treated patients had significantly suppressed progression of ALS symptoms compared with 15 placebo controls. Mild diarrhea occurred in two individuals in each group, but no other side effects were observed. During the study period, three subjects in the placebo control group and one subject in the TUDCA-treated group died (Elia et al., 2016). Obesity causes ER stress, inducing insulin resistance. Ten obese patients, whose BMI was above 35, were treated with 1,750 mg of TUDCA daily for 4 weeks in another clinical trial. In TUDCA-treated patients, the insulin sensitivity in liver and muscle was significantly increased compared with that before treatment; however, no significant change was observed in the 10 patients who received a placebo control. In muscle cells of TUDCA-treated patients, phosphorylation of IRS-1 and Akt (protein kinase B) was enhanced, and insulin receptor signaling was improved. No side effects were reported (Kars et al., 2010).

**Table 1:** Overview of clinical trials using pharmacological chaperones.

Drugs	Diseases	Highlights	Reference
TUDCA	ALS	Slower progression in the TUDCA (1 g twice daily for 54 weeks) than in the placebo group (*P* < 0.01).	Eur J Neurol. 2016 Jan; 23(1): 45–52.
TUDCA	Insulin resistance	Hepatic and muscle insulin sensitivity increased by approximately 30% (P < 0.05) after treatment with TUDCA (1,750 mg/day) for 4 weeks in obese patients.	Diabetes. 2010 Aug;59(8):1899-905.
TUDCA	Endothelial dysfunction	Hyperglycemia-induced endothelial dysfunction can be mitigated by oral administration of TUDCA (1500 mg).	Clin Sci (Lond). 2016 Nov 1; 130(21): 1881–1888.
TUDCA	Transthyretin amyloidosis	Non-progression of the neuropathy and of the cardiomyopathy was obtained by orally doxycycline (100 mg BID) and TUDCA (250 mg 3 times/day) administered continuously for 12 months.	Amyloid. 2012 Jun;19 Suppl 1:34-6.
PBA	ALS	Twenty-six participants with medication from 9 to 21 g/day completed the 20-week treatment phase. PBA was safe and tolerable.	Amyotroph Lateral Scler. 2009 Apr;10(2):99-106.
PBA	insulin resistance	2 weeks of oral PBA (7.5 g/day) reduce ER stress, partially alleviates lipid-induced insulin resistance and β-cell dysfunction in obese humans.	Diabetes. 2011 Mar; 60(3): 918–924.
UDCA	ALS	Oral administration of UDCA at doses of 15, 30, and 50 mg/kg of body weight per day for 4 weeks is well tolerated and crosses the blood-brain barrier in a dose-dependent manner.	Clin Neuropharmacol. Jan-Feb 2010;33(1):17-21.
AMX0035 (TUDCA and PBA)	Alzheimer's Disease	Study to Assess the Safety and Biological Activity of AMX0035 for the Treatment of Alzheimer's Disease.	https://clinicaltrials.gov/
AMX0035	ALS	Assess longer term safety and therapeutic potential of AMX0035 for patients with ALS.	https://clinicaltrials.gov/

Orally administered TUDCA is mostly metabolized in the liver because of entero-hepatic circulation (Kusaczuk, 2019). This may limit its distribution to the systemic circulation in sufficient amounts; therefore, intravenous or subcutaneous administration may be more effective systemically.

## Conclusion

Pharmacological chaperones such as TUDCA are considered to promote proteostasis in infected cells, maintain proper folding of functional proteins, and suppress the progression of inflammation and apoptosis. This may result in the survival of SARS-CoV-2-infected cells, and in turn, the virus. Therefore, by the simultaneous administration of antiviral therapy and pharmacological chaperone therapy in combination, the two modalities may constitute a promising therapeutic strategy for COVID-19.

## Author Contributions**

The author confirms being the sole contributor of this work and has approved it for publication.

## Funding

This work was funded by Grants-in-Aid from the Japan Society for the Promotion of Science (KAKENHI) to TA (17K11114).

## Conflict of Interest

The author declares that the research was conducted in the absence of any commercial or financial relationships that could be construed as a potential conflict of interest.
